# Splenic Artery Pseudoaneurysm Causing Delayed Hematemesis After Total Gastrectomy With D2 Lymphadenectomy

**DOI:** 10.7759/cureus.107264

**Published:** 2026-04-17

**Authors:** Aliki Vaia Rompou, Kleanthi C Ampntin, Ilias Vourlitis, Andreas Nikolaos Dafnis, Giorgos Gkeneralis, Dimitris P Korkolis

**Affiliations:** 1 Surgical Oncology, General Anticancer-Oncology Hospital of Athens "Saint Savvas", Athens, GRC; 2 Interventional Unit of Radiology, General Anticancer-Oncology Hospital of Athens "Saint Savvas", Athens, GRC

**Keywords:** angiography, d2 lymphadenectomy, delayed postoperative hemorrhage, endovascular stent placement, sentinel bleeding, total gastrectomy

## Abstract

Delayed postoperative hemorrhage after total gastrectomy is rare and may occur in the absence of an identifiable intraluminal bleeding source. Vascular complications, including splenic artery pseudoaneurysm, represent an uncommon but potentially life-threatening etiology. We report the case of a 67-year-old woman who underwent open total gastrectomy with Roux-en-Y reconstruction and standard D2 lymphadenectomy for gastric adenocarcinoma. Fourteen days postoperatively, she presented with acute hematemesis and hemodynamic instability. Endoscopic and surgical exploration failed to identify a bleeding source. Computed tomography angiography revealed a splenic artery pseudoaneurysm, which was successfully managed with endovascular stent placement. Splenic artery pseudoaneurysm should be considered in patients presenting with delayed bleeding after gastrectomy when conventional investigations are inconclusive. Computed tomography angiography is essential for diagnosis, and endovascular therapy is a safe and effective first-line treatment.

## Introduction

Postoperative bleeding after gastrectomy is uncommon but potentially life-threatening. While early hemorrhage is usually related to technical factors at staple lines or anastomoses, delayed bleeding is rarer, diagnostically challenging, and may follow an intermittent “sentinel bleeding” pattern [1-3]. In patients with negative upper gastrointestinal endoscopy, other less obvious sources of bleeding should be actively considered [1-4].

Splenic artery pseudoaneurysm is a rare etiology of delayed upper gastrointestinal bleeding and is most often reported in association with pancreatitis, trauma, or surgery involving perigastric/suprapancreatic dissection [5-8]. During oncologic total gastrectomy with D2 lymphadenectomy, dissection along the suprapancreatic vessels and the splenic artery (station 11) may predispose to delayed arterial wall injury (traction, clip/thermal injury) and pseudoaneurysm formation [9,10].

Computed tomography angiography (CTA) has a central role in localizing vascular lesions when conventional investigations are inconclusive [6,11]. Endovascular therapy (embolization or covered stent placement) is widely considered first-line in appropriate patients, with high technical success and lower morbidity compared with reoperation [8,12,13]. We report a case of delayed hematemesis after total gastrectomy with D2 lymphadenectomy due to splenic artery pseudoaneurysm treated successfully with endovascular covered stenting.

## Case presentation

A 67-year-old woman with biopsy-proven gastric adenocarcinoma underwent appropriate staging, which demonstrated no evidence of distant metastatic disease. The tumor was located in the proximal stomach involving the cardia, and histopathological examination of endoscopic biopsy specimens revealed moderately differentiated adenocarcinoma. Preoperative staging classified the tumor as cT3N1M0 according to the Tumor, Node, Metastasis (TNM) classification. The patient received six cycles of neoadjuvant chemotherapy, following which elective surgical resection was planned.

Her medical history included hypertension and dyslipidemia, managed with angiotensin-converting enzyme inhibitors and statins. She had no history of pancreatitis, trauma, or previous upper abdominal surgery. The patient underwent open total gastrectomy with Roux-en-Y esophagojejunal reconstruction and standard D2 lymphadenectomy in accordance with Japanese Gastric Cancer Association guidelines.

Surgery was performed through an upper midline laparotomy. After systematic abdominal exploration, the greater omentum was divided and the gastrocolic ligament was opened to enter the lesser sac, exposing the anterior surface of the pancreas. A formal D2 lymphadenectomy was undertaken, including removal of perigastric lymph nodes (stations 1-6) and suprapancreatic nodes along the left gastric artery, common hepatic artery, celiac trunk, and splenic artery (stations 7-11), as well as station 12a along the proper hepatic artery.

The right gastroepiploic vessels were ligated at their origin, followed by division of the right gastric artery. The duodenum was transected distal to the pylorus using a linear stapling device. The left gastric artery was divided at its origin with en bloc lymphadenectomy. Suprapancreatic dissection along the splenic artery (station 11) was performed with careful skeletonization of the vessel.

Vascular ligation was achieved using clips and non-absorbable sutures. Lymphadenectomy was performed with a combination of bipolar energy devices and ultrasonic shears. Reconstruction was completed with a circular stapled end-to-side esophagojejunal anastomosis in a Roux-en-Y configuration.

The operative time was approximately 180 minutes, with an estimated blood loss of 250 mL. The immediate postoperative course was uneventful, and the patient was discharged in stable condition. On postoperative day 14, she presented with acute hematemesis and hypovolemic symptoms. Laboratory tests demonstrated a significant drop in hemoglobin. She was resuscitated and transfused, achieving temporary stabilization.

Following the initial episode, cross-sectional imaging has been considered. However, the patient developed recurrent massive hematemesis with rapid hemodynamic deterioration. In this setting, transfer for computed tomography angiography (CTA) was deemed unsafe due to the risk of cardiovascular collapse. Emergency upper gastrointestinal endoscopy was performed but failed to identify an active bleeding source, and the esophagojejunal anastomosis appeared intact.

**Figure 1:**
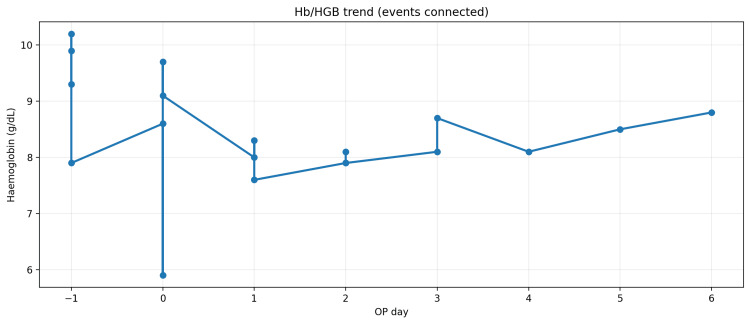
Hemoglobin trend from readmission to postoperative day 6 (operative date is day 0) Serial hemoglobin (Hb) measurements demonstrating a progressive decline associated with episodes of delayed postoperative hemorrhage, followed by stabilization after endovascular treatment. OP: operative, HGB: hemoglobin.

Given ongoing hemodynamic instability and the absence of an identifiable intraluminal source, urgent exploratory surgery was undertaken. Despite thorough intraoperative inspection and evacuation of a significant volume of blood, no bleeding source was identified. The patient subsequently achieved transient hemodynamic stabilization. Angiographic evaluation performed thereafter demonstrated a 2 cm pseudoaneurysm arising from the mid-segment of the splenic artery (Figure 1), without evidence of active contrast extravasation.

**Figure 2:**
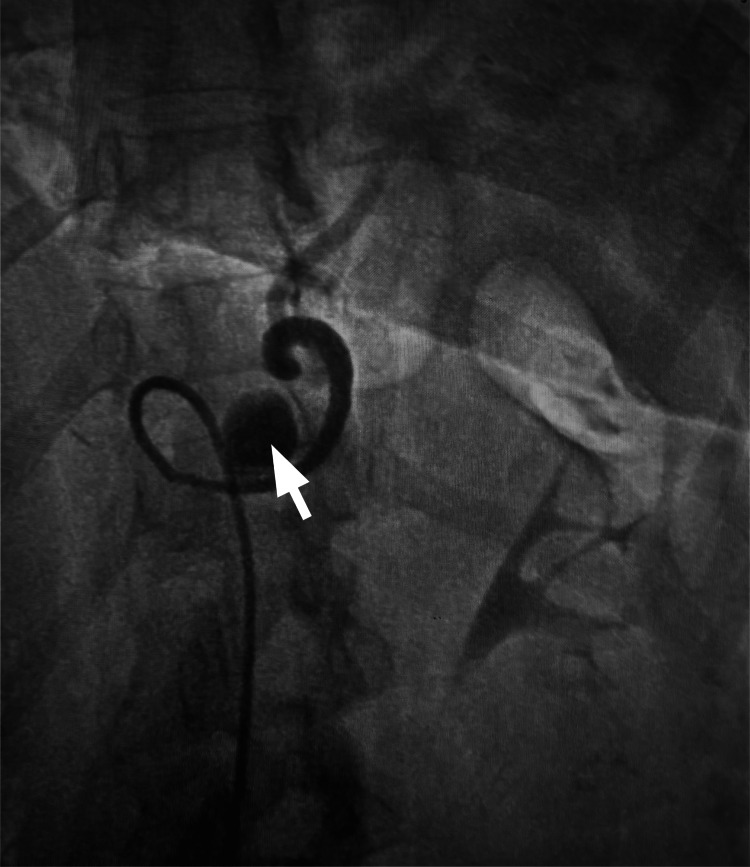
Selective splenic artery angiography demonstrating pseudoaneurysm Selective angiography of the splenic artery demonstrates a focal contrast-filled outpouching (arrow) consistent with a pseudoaneurysm arising from the mid-segment of the splenic artery.

**Figure 3:**
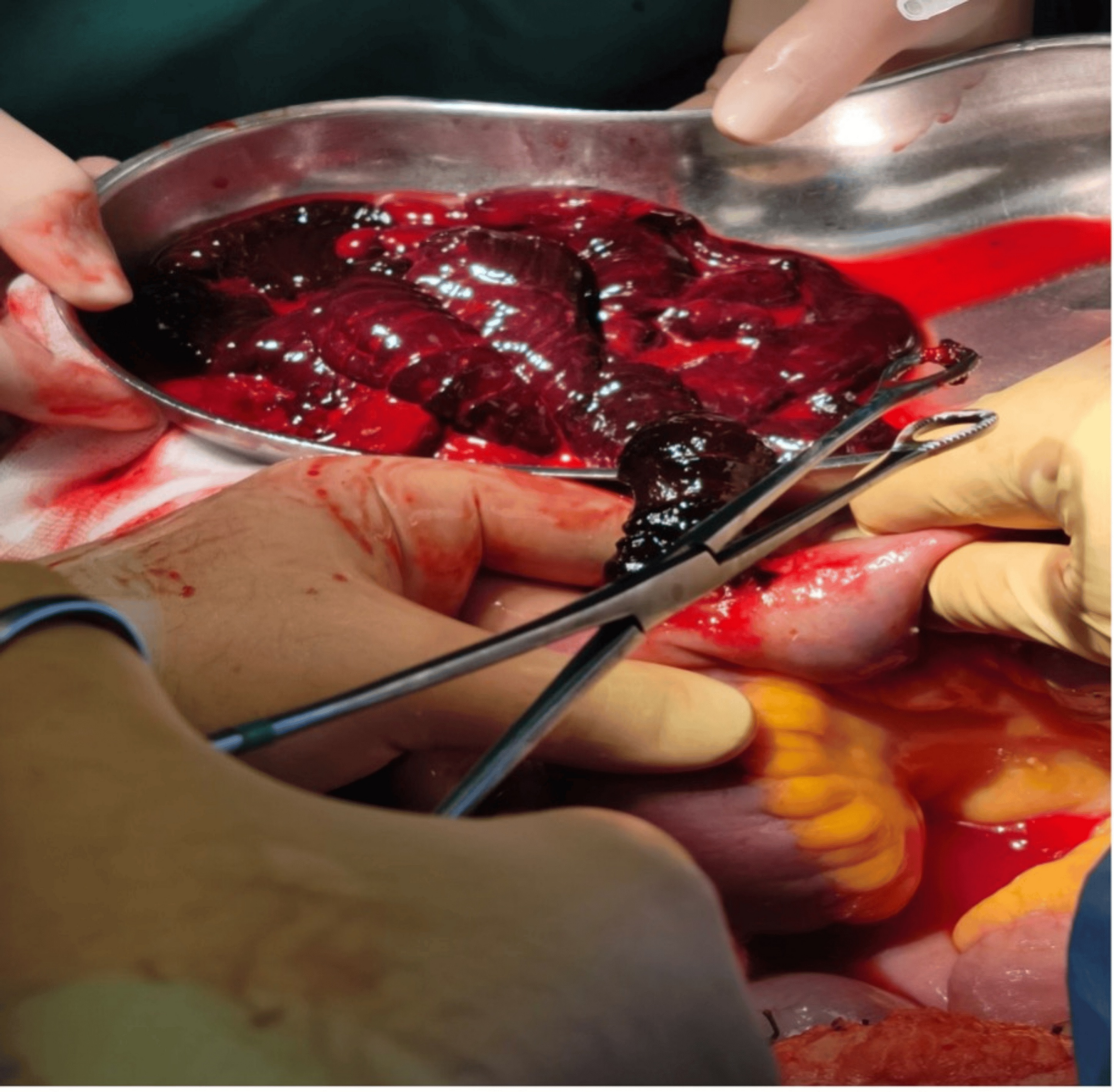
Intraoperative findings during exploratory laparotomy Significant intraluminal blood clots were observed; however, no active bleeding source was identified despite thorough inspection.

The pseudoaneurysm was considered the most likely source of intermittent hemorrhage. In this context, the source of bleeding is likely related to intermittent communication between the splenic artery pseudoaneurysm and the adjacent small bowel, most plausibly the Roux limb or a nearby jejunal loop. This may occur through focal erosion or inflammatory adherence between the arterial wall and the bowel, allowing episodic leakage of blood into the intestinal lumen. Such a mechanism explains the presence of significant intraluminal clot, the absence of active bleeding during endoscopic and intraoperative assessment, and the characteristic intermittent or “sentinel bleeding” pattern observed in this case. The patient underwent successful endovascular treatment with covered stent placement, resulting in immediate clinical stabilization (Figures 2, 3). No further bleeding episodes occurred. The total hospital stay was six days. At follow-up of six months, the patient remained asymptomatic with no recurrence of bleeding.

## Discussion

Delayed postoperative hemorrhage following gastrectomy is an uncommon but potentially life-threatening complication [1-3]. While early bleeding is typically related to technical factors such as anastomotic or staple-line failure, delayed hemorrhage is less frequent and often associated with vascular complications, including arterial erosion and pseudoaneurysm formation [1,2]. These delayed presentations may follow an intermittent or “sentinel bleeding” pattern, characterized by self-limited episodes preceding catastrophic hemorrhage, which can complicate timely diagnosis [1-4].

Splenic artery pseudoaneurysm (SAP) is a rare but well-recognized cause of massive upper gastrointestinal bleeding [5,6]. It is most commonly associated with pancreatitis, trauma, or pancreatic surgery; however, it has also been described following upper gastrointestinal oncologic procedures, including gastrectomy [5-8]. During total gastrectomy with D2 lymphadenectomy, dissection along the suprapancreatic vessels necessitates skeletonization of the splenic artery (station 11), which may predispose to delayed vascular injury [9,10]. Even in the absence of overt intraoperative bleeding, subtle arterial wall damage, resulting from traction, clip application, or thermal spread from energy devices, may lead to progressive weakening of the vessel wall and subsequent pseudoaneurysm formation [5-8].

Clinically, SAP may present with hematemesis or melena, often preceded by intermittent bleeding episodes [1,3]. When bleeding arises from an extraluminal arterial lesion that intermittently communicates with the gastrointestinal tract, standard diagnostic modalities such as upper endoscopy may fail to identify an active source [1-4]. In the present case, the absence of identifiable bleeding on both endoscopic and surgical exploration highlights this diagnostic limitation and underscores the need for a high index of suspicion for vascular etiologies.

The mechanism of bleeding in this case is most likely related to intermittent communication between the pseudoaneurysm and the gastrointestinal lumen, potentially through erosion into adjacent jejunal loops or the esophagojejunal anastomosis. This intermittent communication explains the episodic nature of hemorrhage, the presence of significant intraluminal blood, and the absence of active bleeding during endoscopic and intraoperative assessment.

This case further highlights a critical aspect of clinical decision-making in the management of postoperative hemorrhage: the timing of diagnostic imaging. In patients presenting with delayed bleeding after gastrectomy, particularly when initial endoscopic evaluation is negative, early consideration of vascular causes is essential [1-4]. A key learning point is the existence of a transient “diagnostic window” during early hemodynamic stabilization, during which computed tomography angiography (CTA) may be safely performed and can facilitate early diagnosis.

In the present case, although the patient achieved temporary stabilization following initial resuscitation, rapid clinical deterioration with recurrent massive hematemesis necessitated immediate intervention. At that stage, transfer for CTA was considered unsafe due to the risk of cardiovascular collapse, and urgent surgical exploration was performed in accordance with emergency management principles. However, retrospectively, earlier use of CTA during the initial stabilization phase may have enabled prompt identification of the vascular lesion and potentially avoided reoperation. In addition, system-related factors, including limited immediate access to advanced imaging in emergency settings, may further influence clinical decision-making in such scenarios.

CTA plays a pivotal role in the diagnosis of visceral artery pseudoaneurysms, allowing rapid identification of focal contrast-filled outpouchings, accurate delineation of vascular anatomy, and appropriate planning of therapeutic intervention [6,11]. A structured diagnostic approach incorporating early CTA following negative endoscopy may therefore improve outcomes in similar cases. Endovascular therapy has become the preferred first-line treatment for visceral artery pseudoaneurysms, particularly in hemodynamically stable or stabilized patients [8,12]. Both coil embolization and covered stent placement are effective treatment modalities. While embolization may result in compromised splenic perfusion and carries a risk of infarction, covered stent placement offers the advantage of excluding the pseudoaneurysm while preserving arterial flow, thereby minimizing ischemic complications [8,12]. In this case, endovascular stent placement resulted in immediate hemodynamic stabilization and complete resolution of bleeding. Overall, this case emphasizes the importance of maintaining a high index of suspicion for vascular injury in patients presenting with delayed hematemesis following gastrectomy. It also highlights the limitations of conventional diagnostic approaches and reinforces the role of early imaging and minimally invasive endovascular management in improving patient outcomes.

## Conclusions

Splenic artery pseudoaneurysm is a rare but potentially catastrophic cause of delayed hemorrhage after total gastrectomy with D2 lymphadenectomy. When endoscopy and surgical exploration fail to identify a bleeding source, computed tomography angiography should be promptly performed. Endovascular treatment is safe and effective and should be considered the treatment of choice in appropriate patients.
